# Soil Phosphorus Conditions Drives Arbuscular Mycorrhizal Fungal Community Differentiation and Modulate the Growth of *Cenchrus pauciflorus*

**DOI:** 10.3390/plants15203085

**Published:** 2026-10-09

**Authors:** Xun Su, Wenjing Ma, Guangchao Lyu, Zhichao Xiao, Yi Qi, Jia Li, Jiahui Qi, Yujie Chen, Xun Tian

**Affiliations:** College of Life Sciences and Food Engineering, Inner Mongolia Minzu University, Tongliao 028000, China; xuesun2311@126.com (X.S.);

**Keywords:** *Cenchrus pauciflorus*, arbuscular mycorrhizal fungi, soil phosphorus, community composition, symbiotic effect, invasion mechanism

## Abstract

*Cenchrus pauciflorus* is a major invasive plant in the sandy lands of northern China, with the Horqin Sandy Land being a hotspot of its invasion, where soil phosphorus availability may influence its spread through arbuscular mycorrhizal fungi (AMF). To elucidate the effects of soil phosphorus on AMF communities and their role in the invasion of *C. pauciflorus*, we conducted a field survey at two sites with contrasting phosphorus backgrounds: Chajintai Ranch (high phosphorus) and Bahuta Sumu (low phosphorus). Rhizosphere soil and root samples of *C. pauciflorus* and the co-occurring native grass *Setaria viridis* were collected for high-throughput sequencing of 18S SSU rRNA gene to analyze AMF community composition and diversity. Two AMF species, *Funneliformis mosseae* and *Diversispora epigaea*, were selected for a pot experiment with *C. pauciflorus*. Five phosphorus treatments were established under high- and low-phosphorus conditions using different phosphorus forms. The effects of the two AMF species on the growth and reproduction of *C. pauciflorus* under different phosphorus treatments were analyzed. The results showed that Glomeraceae and *Glomus* were the predominant family and genus, respectively. Under Bahuta Sumu, *Diversispora* was relatively enriched in the roots of *C. pauciflorus* compared with *S. viridis* roots. However, other nutrient factors also varied between the two field sites, the independent effect of phosphorus on AMF community composition and diversity could not be fully disentangled. The pot experiment revealed that AMF inoculation did not significantly promote the growth of *C. pauciflorus* under high-phosphorus treatment. In contrast, when phosphorus was low and supplied in insoluble forms, both AMF species significantly promoted the growth and reproduction of *C. pauciflorus*. *D. epigaea* exhibited a stronger growth-promoting effect than *F. mosseae*, with significantly higher colonization rates in most treatments. In summary, *C. pauciflorus* is associated with the enrichment of specific AMF taxa in low-phosphorus habitats, which may in turn promote its growth by enhancing the acquisition of insoluble phosphorus. This may represent an important belowground symbiotic mechanism underlying its successful invasion in nutrient-poor sandy habitats.

## 1. Introduction

Arbuscular mycorrhizal fungi (AMF) are among the most important plant symbiotic microbial groups in terrestrial ecosystems. They play a key role in the acquisition of phosphorus, nitrogen, and other nutrients by host plant, and profoundly influence plant ecological adaptability and community assembly processes by affecting root architecture, photosynthetic product allocation, stress resistance, and competitive interactions [1,2]. Soil phosphorus occurs in various forms, including soluble inorganic phosphorus, iron- and aluminum-bound phosphorus, calcium-bound phosphorus, and organic phosphorus. The availability of different phosphorus forms to plants varies considerably. In low-phosphorus or nutrient-heterogeneous habitats, AMF generally enhance the acquisition of sparingly soluble phosphorus by host plant, thereby promoting plant growth and reproductive output. In high-phosphorus environments, however, host plant dependence on AMF declines, leading to reduced AMF colonization, and diminished symbiotic benefits. For example, AMF colonization rate is negatively correlated with soil inorganic phosphorus and available phosphorus concentrations [3,4]. Therefore, soil available phosphorus level and phosphorus forms not only are regulate AMF colonization and community composition but also modulate the symbiotic effects of AMF on host plants, thereby influencing plant nutrient acquisition, growth, and competitive ability [5,6]. Nevertheless, in invasive plants, it remains unclear how soil phosphorus availability and phosphorus speciation affect AMF communities and their symbiotic effects on hosts.

For invasive plants, soil phosphorus availability not only affects plant growth but also influence invasion processes by altering rhizosphere AMF communities. An increasing number of studies have shown that certain invasive plants can enhance rhizosphere nutrient acquisition by selectively recruiting beneficial AMF taxa and influencing the composition and function of soil microbial communities, thereby gaining competitive advantages in novel environments [7,8]. For example, the invasive plant *Triadica sebifera* markedly promotes AMF colonization by enhancing the biosynthesis and exudation of strigolactones and flavonoids, thereby gaining an competition advantage in nutrient acquisition [9]. The classic “enhanced mutualism” hypothesis proposes that invasive plants in newly colonized regions may promote their growth and enhance adaptation to resource-limited environments by utilizing native AMF more efficiently [10,11]. In addition, altered soil microbial communities induced by invasive plants may in turn favor their growth, forming a positive plant–soil feedback [12]. However, different AMF taxa vary in their responses to hosts and environments. Some AMF taxa tend to perform better under low-phosphorus conditions or in the presence of specific phosphorus forms (such as sparingly soluble inorganic phosphorus or organic phosphorus), while others are better adapted to higher nutrient availability or specific pH and organic matter conditions [13,14]. Overall, soil phosphorus may modulate host plant root exudates, influence the selective recruitment of rhizosphere AMF, and subsequently alter plant nutrient acquisition and competitive performance. Under different phosphorus conditions, invasive plants and co-occurring native plants may recruit AMF communities of different compositions, thereby affecting their the functional outcomes of these AMF communities on host plants. Nevertheless, how such compositional differences vary with phosphorus conditions and whether these differences further influence the growth of invasive plants remains unclear.

*Cenchrus pauciflorus* is one of the most dangerous invasive plants in grassland and sandyland ecosystems in northern China. It is characterized by high reproductive capacity, broad adaptability, and rapid spread, posing a serious threat to grassland productivity and community stability [15]. Previous studies have shown that *C. pauciflorus* can grow rapidly and form monodominant communities in phosphorus-limited and nutrient-poor sandy soils. Its successful invasion may depend not only on the plasticity of its resource acquisition strategies but also on synergistic phosphorus acquisition mediated by rhizosphere microorganisms, particularly AMF. Marked spatial variation in soil phosphorus supply exists across the Horqin Sandy Land, providing suitable conditions for comparing interactions between *C. pauciflorus* and AMF under contrasting phosphorus conditions. On this basis, the present study selected two sites with different phosphorus conditions in the Horqin Sandy Land, using *C. pauciflorus* and the co-occurring species *Setaria viridis* as comparison species. By combining high-throughput sequencing of AMF in roots and rhizosphere soil with pot inoculation experiments, this study aimed to: (1) reveal differences in rhizosphere AMF community composition between *C. pauciflorus* and *S. viridis* under different soil phosphorus conditions; (2) identify AMF taxa specifically enriched by *C. pauciflorus*; and (3) compare the growth- and biomass-promoting effects of different AMF species on *C. pauciflorus*. The results are expected to reveal the phosphorus adaptation strategies of *C. pauciflorus* from the dual perspectives of AMF community differentiation and functional validation, and to provide a theoretical basis for the microbial management of invasive plants in sandy land ecosystems.

## 2. Results

### 2.1. AMF Community Composition and α-Diversity in the Rhizosphere and Roots of Cenchrus pauciflorus and Setaria viridis

In this study, total phosphorus (TP) and available phosphorus (AP) at Chajintai Ranch were significantly higher than those at Bahuta Sumu (Table 1), providing suitable conditions for comparing AMF community variation under different phosphorus conditions. Sample codes rules are defined as follows: C, Chajintai Ranch: B, Bahuta Sumu; S, *Cenchrus pauciflorus*: G, *Setaria viridis*; the second S, rhizosphere soil; and R, roots. For example, CSS denotes rhizosphere soil of *C. pauciflorus* from Chajintai Ranch, CRS denotes roots of *C. pauciflorus* from Chajintai Ranch, BSG denotes rhizosphere soil of *S. viridis* from Bahuta Sumu, and BRG denotes roots of *S. viridis* from Bahuta Sumu. All sample codes in this study follow this naming scheme.

A total of 475 AMF OTUs were obtained from 48 samples after removing chimeras and clustering non-repeated sequences at 97% similarity, excluding singletons. These OTUs belonged to 1 phylum, 4 classes, 6 orders, 11 families, 12 genera, and 68 species. The Venn diagram (Figure 1A) showed that the eight sample groups (BRS, CRS, BSG, CSG, BSS, CSS, BRG and CRG) shared 33 AMF OTUs. The numbers of unique OTUs in each group were 18, 15, 29, 37, 5, 49, 9, and 4, respectively. CSS and CSG had the highest numbers of OTUs, indicating that AMF richness in the rhizosphere soil of both plant species was higher at Chajintai Ranch than at Bahuta Sumu.

α-Diversity analysis (Figure 1B–E) showed significant differences in AMF α-diversity among eight sample groups (*p* < 0.05). The Ace and Chao indices of CSG and CSS were significantly higher than those of all other groups (*p* < 0.05). The α-diversity of rhizosphere soil samples BSG, CSG, and CSS was significantly higher than that of their corresponding root samples (*p* < 0.05). The Shannon index differed significantly among groups, but pairwise comparisons revealed significant differences only between BSG and BRG, and between CSS and CRS (*p* < 0.05). The Simpson index was highest value in BRG, with no significant differences among the other sample groups (*p* > 0.05). Overall, AMF α-diversity was higher in rhizosphere soil than in roots, but no significant differences were found between *S. viridis* and *C. pauciflorus* within the same sample type.

At the family level (Figure 1F), 11 families were detected, among which Glomeraceae was the absolute dominant family, showing no significant difference between *S. viridis* and *C. pauciflorus*. The top five most abundant AMF families across all sample groups were Glomeraceae, Diversisporaceae, Acaulosporaceae, Claroideoglomeraceae, and Gigasporaceae. Among them the relative abundances of Diversisporaceae and Acaulosporaceae were higher in BRS (roots of *C. pauciflorus* at Bahuta Sumu) than in other sample groups, suggesting that the root AMF community of *C. pauciflorus* at Bahuta Sumu differed from that of *S. viridis* at the family level. At the genus level (Figure 1G), 12 genera were detected, with *Glomus* being the dominant genus. The five most abundant genera were *Glomus*, *Diversispora*, *Acaulospora*, *Claroideoglomus*, and *Gigaspora*. The relative abundances of *Diversispora* and *Acaulospora* were higher in BRS than in the other sample groups. These results indicate that the root AMF community of *C. pauciflorus* at Bahuta Sumu differed from that of *S. viridis* at the same at both family and genus levels.

### 2.2. Analysis of AMF Community Structure

NMDS analysis (Figure 2A) showed that the four sample groups from Bahuta Sumu exhibited a relatively clustered AMF community distribution, with a stress value of 0.136 in two-dimensional ordination. ANOSIM analysis indicated no significant difference among the four groups (*R* = 0.059, *p* = 0.199), suggesting high similarity in AMF community composition between *C. pauciflorus* and *S. viridis* at this site. In contrast, the four sample groups from Chajintai Ranch (CSS, CSG, CRS, and CRG) were more dispersed, with clear separation between rhizosphere and root samples, whereas differentiation between *C. pauciflorus* and *S. viridis* was relatively weak within each sample type. ANOSIM analysis (Figure 2B) showed significant differences among these four groups (*R* = 0.643, *p* = 0.001), indicating higher heterogeneity of AMF community composition at Chajintai Ranch than at Bahuta Sumu.

### 2.3. AMF Colonization Rates in Cenchrus pauciflorus Roots Under Different AMF Inoculation and Phosphorus Treatments

Microscopic examination showed that no vesicles or hyphae in the roots of non-inoculated controls (Figure 3A–E). In contrast, typical vesicles and hyphal colonization were observed in roots inoculated with *Funneliformis mosseae* (Fm) and *Diversispora epigaea* (De) (Figure 3F–O), indicating successful inoculation and no AMF contamination occurred in the controls. Vesicle and hyphal colonization rates differed between Fm and De under different phosphorus treatments. For vesicle colonization rate, De showed significantly higher values than Fm under HP and LAl treatments (*p* < 0.05), while no significant differences were observed under LP, LZ, and LQ treatments (*p* > 0.05). For hyphal colonization rate, De showed significantly values than Fm under LP and LQ treatments (*p* < 0.05), but significantly lower values than Fm under LAl treatment (*p* < 0.05). No significant differences were found between Fm and De under the remaining treatments (*p* > 0.05).

### 2.4. Effects of AMF Inoculation and Phosphorus Treatments on the Growth of Cenchrus pauciflorus

AMF inoculation and AMF species identity had different effects on the growth of *C. pauciflorus* under the different phosphorus treatments (Figure 4A–E), and growth indices also differed among treatments (Figure 4F–K). Under HP treatment, plant height, green leaf number, withered leaf number, spike number, and fruit number in AMF-inoculated groups were all greater than those in the CK group. Only plant height under the De treatment and spike number under the Fm treatment were significantly higher than in CK (*p* < 0.05). Under the LP treatment, the growth-promoting effect of AMF was less pronounced; only green leaf number of and spike number were significantly higher in both AMF treatments than those in CK (*p* < 0.05). Under the LAl treatment, green leaf number, leaf area, and fruit number followed the order De > Fm > CK, with De treatment being significantly higher than CK (*p* < 0.05). Under the LZ treatment, plant height, leaf area, and fruit number in both AMF treatments were significantly greater than those in CK (*p* < 0.05). Under the LQ treatment, except for withered leaf number and spike number, all other indices in the De treatment were significantly greater than in CK (*p* < 0.05).

Under the HP treatment, root, stem, green leaf, and total biomass of non-inoculated *C. pauciflorus* were all higher than those in the AMF-inoculated treatments, indicating that inoculation with either AMF species had no positive effect on biomass accumulation under high phosphorus conditions. Under the LP treatment, root biomass, green leaf biomass, and total biomass were highest in the Fm treatment and significantly greater than those in CK (*p* < 0.05), whereas fruit biomass was highest in the De treatment and significantly greater than that in CK. Under the LAl treatment, all organs biomass and total biomass followed the order De > Fm > CK; except for withered leaf biomass, all indices in the De treatment were significantly higher than those in CK (*p* < 0.05). Under the LZ treatment, root, stem, withered leaf, and total biomass were highest in the Fm treatment, with only stem biomass being significantly greater than that in CK (*p* < 0.05). Under the LQ treatment, stem, green leaf, and fruit biomass, as well as total biomass in the De treatment were significantly greater than those in CK (*p* < 0.05), and green leaf biomass and fruit biomass were significantly higher than those in the Fm treatment (*p* < 0.05) (Figure 5).

## 3. Discussion

### 3.1. Differences in AMF Community Composition and Diversity Under Different Phosphorus Conditions

The two sites (Chajintai Ranch and Bahuta Sumu) differed significantly in soil physicochemical properties. TP, TN, AK, AN, and AP were all significantly higher at Chajintai Ranch than at Bahuta Sumu, indicating distinct nutrient supply conditions and providing suitable conditions for comparing AMF community responses under contrasting phosphorus conditions.

Soil available phosphorus level and phosphorus speciation are considered important drivers of AMF community structure [14,16]. AMF α-diversity analysis indicated that AMF OTU richness and Ace, Chao, and Shannon indices were generally higher at Chajintai Ranch than at Bahuta Sumu. The Ace and Chao indices of BSG, CSG, and CSS were significantly higher than those of their corresponding root samples. This result is consistent with the more open nature of the rhizosphere environment compared with the root interior, which can accommodate more opportunistic and generalist AMF taxa. Studies have shown that AMF OTU richness in soil is significantly higher than that in roots (e.g., 54 vs. 34 OTUs), and that soil and root AMF communities differ markedly. Root AMF communities are subject to host selective filtering, while soil communities more directly reflect soil environmental conditions [17]. It should be noted that the two sites not only differed in phosphorus availability but also exhibited synchronous changes in other soil properties. Therefore, AMF community differences between the two field sites resulted from the combined effects of multiple soil factors, and the independent effect of phosphorus could not be fully disentangled at the field level. This study focused on the response characteristics of AMF communities under different phosphorus conditions; the independent effect of phosphorus will be further examined through subsequent controlled experiments.

Notably, Glomeraceae was the dominant family and *Glomus* was the most dominant genus across all samples. This is consistent with the widespread global distribution, broad ecological tolerance, and high adaptability of this genus to disturbance and nutrient fluctuations [2]. As a typical broad-niche AMF family, Glomeraceae can maintain high abundance under various soil conditions and often dominates in pioneer and herbaceous plants and in disturbed habitats [18]. These results suggest that despite differences in phosphorus conditions and other nutrient conditions between the two sites, broad-niche taxa still dominate the AMF community, indicating a certain degree of structural conservatism.

NMDS analysis showed that the four sample groups from Bahuta Sumu had relatively clustered AMF community distribution, indicating minor differences in AMF community composition among hosts and sample types at this site. In contrast, rhizosphere soil and root samples from Chajintai Ranch were more dispersed, with clear separation between rhizosphere soil and root samples, indicating higher heterogeneity of AMF community composition at this site than at Bahuta Sumu. The greater dispersion may reflect higher environmental heterogeneity at Chajintai Ranch, whereas the clear separation between rhizosphere soil and root samples may reflect stronger host selection. However, due to the observational nature of this field study, the relative contributions of individual soil factors could not be fully distinguished. Frew et al. found through controlled experiments that increasing soil phosphorus availability reduced the phylogenetic diversity of AMF communities, with increased phylogenetic clustering. This suggesting that under high phosphorus, host plants exert stronger selective pressure on fungal functional groups, with selection, shifting from nutrient acquisition to defense functions [19]. In the present study, however, the greater dispersion of AMF communities at the high-phosphorus site (Chajintai Ranch) reflects variation in beta diversity among samples and therefore is not directly comparable to the phylogenetic clustering reported by Frew et al. Whether host selection increases under high-phosphorus conditions still needs to be verified using host performance and AMF functional validation. Importantly, despite the overall differences in AMF communities, whether *C. pauciflorus* and *S. viridis* exhibit host-specific AMF community differentiation is key to understanding the phosphorus adaptation strategies of the invasive species.

### 3.2. Ecological Significance of AMF Community Differentiation Between Cenchrus pauciflorus and Setaria viridis

The relative abundances of *Diversispora* and *Acaulospora* were higher in the roots of *C. pauciflorus* at Bahuta Sumu (BRS group) than in *S. viridis* roots at the same site, suggesting that *C. pauciflorus* may selectively enrich specific AMF taxa under low-nutrient conditions. It should be noted that the field data in this study only reveal differences in community composition; whether *C. pauciflorus* actively “recruits” these taxa requires further validation through root exudate analysis or inoculation experiments. Such host-associated differences in AMF communities has also been reported in other invasive plants. For example, the invasive plant *Solidago canadensis* can associate with different AMF communities compared with its native congener, and this difference is further amplified when soil phosphorus speciation is altered [16].

Different AMF taxa differ in resource acquisition strategies. Studies have shown that AMF functional traits are related to their resource acquisition strategies. “Fast-growing” taxa (e.g., *Glomus*) tend to proliferate in nutrient-rich or disturbed environments [20], whereas some large-spored, low-dispersal taxa are often associated with nutrient-poor soils or stable host environments [21]. The enrichment of *Diversispora* and *Acaulospora* in BRS may reflect an adaptive strategy by which *C. pauciflorus* to compensate for low nutrient supply by preferentially enriching high-return AMF taxa.

Such preferential enrichment may result from the regulated release of specific signaling molecules (such as strigolactones and flavonoids) in root exudates of *C. pauciflorus*. These signaling molecules are typically released under low-phosphorus conditions to promote symbiotic association with specific AMF taxa [22]. From an invasion ecology perspective, the preferential enrichment of specific AMF taxa by *C. pauciflorus* supports the “enhanced mutualism” hypothesis, which suggests that invasive plants may enhance their adaptation to resource-limited environments by using native AMF more efficiently in newly colonized areas. Chen et al. found that soil phosphorus speciation significantly altered the extent of differences in AMF communities between the invasive plant *S. canadensis* and its native congener, and that AMF α-diversity and key taxa in the co-occurrence network were the main factors influencing the growth advantage of the invasive plant [16]. Based on these field findings, this study further selected *Funneliformis mosseae* (a representative of the genus *Glomus,* which was significantly enriched by both plants) and *Diversispora epigaea* (a representative of the genus *Diversispora,* which was significantly enriched by *C. pauciflorus*) as AMF inoculants to verify their growth-promoting effects on *C. pauciflorus* under insoluble phosphorus treatments.

### 3.3. Growth-Promoting Effects of Different AMF Species on Cenchrus pauciflorus and Their Dependence on Phosphorus Treatments

The pot experiment revealed that the growth-promoting effects of *Funneliformis mosseae* and *Diversispora epigaea* on *C. pauciflorus* were species-specific and dependent on the phosphorus source. Under the high-phosphorus (HP) treatment, AMF inoculation did not promote growth; several growth indices (leaf area and root biomass in Fm, and spike number, stem biomass, and fruit biomass in De) were significantly lower than those in the non-inoculated control. This result is consistent with the “context-dependent symbiotic mutualism” hypothesis, which states that when external phosphorus supply is sufficient, plants reduce investment in the mycorrhizal pathway, and the symbiotic relationship may shift from mutualism to neutrality or even weak parasitism [23]. Smith and Read emphasized that the relationship between AMF and host plants is strongly influenced by host phosphorus status, which in turn is affected by soil phosphorus availability [24]. Salmerón-Santiago et al. also found in tomato plants that AMF colonization was markedly reduced under high phosphorus, and inoculated and non-inoculated plants showed similar growth, whereas inoculated plants responsed positively under low phosphorus [25].

In contrast, under low-phosphorus treatments, especially aluminum phosphate (LAl), phytic acid (LZ), and hydroxyapatite (LQ), both AMF species promoted plant height, green leaf number, fruit number, and biomass accumulation in all organs of *C. pauciflorus*. This indicates that AMF can help host plants utilize different forms of sparingly soluble phosphorus, ultimately enhancing vegetative growth and reproductive output [26,27]. Notably, *D. epigaea* generally exhibited stronger growth-promoting effects than *F. mosseae* under the LAl and LQ treatments, with particularly pronounced differences in biomass and reproductive indices, indicating that different AMF species are not functionally equivalent and that their contributions to the host differ among species [28].

Colonization rate measurements provide a partial mechanistic explanation for these differences. The vesicle colonization rate of *D. epigaea* was significantly higher than that of *F. mosseae* under the HP, LAl, and LQ treatments, and its hyphal colonization rate was also significantly higher under the LP treatment. Higher colonization rates suggest a more extensive hyphal network and more efficient nutrient uptake, which partly explain the stronger growth-promoting effect of *D. epigaea* under specific phosphorus treatments. The mechanisms underlying functional differences among AMF may involve multiple levels of regulation. Previous studies have shown that different AMF taxa differ in extraradical hyphal extension and soil exploration capacity. Members of *Diversispora* may possess more extensive hyphal networks and higher soil phosphorus capture efficiency [29]. Different AMF species may also differ in their ability to mineralize organic phosphorus or solubilize sparingly soluble inorganic phosphorus [29]. AMF species exhibit significant differences in their phosphorus acquisition strategies, which are closely related to hyphal growth rate and carbon consumption efficiency [30,31]. Even within the same AMF species, different isolates exhibit high functional diversity in phosphate and nitrogen uptake as well as fungal phosphate metabolism [32]. Therefore, the stronger promotion of *C. pauciflorus* growth by *D. epigaea* under insoluble phosphorus treatments may stem from its advantages in hyphal extension capacity, phosphorus mobilization efficiency, or host signaling. However, these traits were not directly measured in the present study, and the underlying mechanisms require further verification.

Combining field and pot results, this study found that *Diversispora* was relatively enriched in the roots of *C. pauciflorus* at Bahuta Sumu. The present study used *D. epigaea* as a representative of the genus *Diversispora* in the inoculation experiment and verified its growth-promoting effect on *C. pauciflorus* under insoluble phosphorus treatments. These results suggest that the stronger response of *C. pauciflorus* to *Diversispora* under different phosphorus forms may provide this species with an underground advantage for expansion in low-nutrient patches in sandy lands.

### 3.4. Limitations and Future Directions

This study revealed the effects of soil phosphorus conditions on AMF communities and the growth of *C. pauciflorus* by integrating community differentiation and functional validation. However, several limitations remain. The field survey only included two sites. Although the soil phosphorus conditions differed significantly, the limited number of sampling sites makes it difficult to fully capture the patterns of AMF community variation between *C. pauciflorus* and co-occurring plants across broader phosphorus gradients. This also restricts inferences regarding the independent effect of phosphorus. The pot experiment used a sterilized sand–vermiculite substrate, which, while ensuring controlled conditions, excluded the influence of other native soil microorganisms (such as bacteria, other fungi, and nematodes). This may have underestimated the regulatory role of interactions among multiple microbial groups in symbiotic effects. In addition, whether the superior growth-promoting effect of *D. epigaea* over *F. mosseae* under insoluble phosphorus treatments results from differences in hyphal phosphatase activity, organic acid secretion characteristics, or phosphorus transporter gene expression remains unresolved. The symbiotic effect under competitive conditions was also not assessed. Whether invasive plants can suppress native plants by monopolizing advantageous AMF resources or altering rhizosphere phosphorus transformation still needs to be verified through competition experiments [16]. Furthermore, the long-term ecological effects of AMF inoculants remain unclear. Whether short-term growth promotion is accompanied by disturbance to the diversity and network stability of native AMF communities requires evaluation through multi-year field trials [19].

Future research can be pursued in three aspects: first, expanding the sampling spatial scale of sampling to include more sites across phosphorus gradients and incorporating long-term monitoring to reveal the responses of AMF communities to dynamic changes in soil phosphorus; second, verifying the core findings in complex soil microcosms or in-situ field controlled experiments to evaluate the regulatory effects of interactions among multiple microbial groups on AMF–host symbiotic associations, and conducting pairwise competition experiments to test the contribution of AMF to the competitive advantage of invasive plants; third, combining transcriptomics and metabolomics to elucidate functional differences among different AMF species in sparingly soluble phosphorus activation pathways, thereby providing a basis for the targeted selection of AMF species in mycorrhizal applications.

## 4. Materials and Methods

### 4.1. Sample Collection

In October 2025, two sites with different phosphorus conditions were selected in the Horqin Sandy Land, Tongliao City, Inner Mongolia, China. Bahuta Sumu (122°14′46.33″ E, 43°14′51.58″ N) is sandy land with aeolian sandy soil, while Chajintai Ranch (122°5′35.28″ E, 43°22′22.50″ N) is degraded grassland with meadow soil. The mean annual precipitation at both sites is 380–450 mm, and the straight-line distance between them is approximately 18 km. The two sites differed significantly in soil physicochemical properties (Table 1). TP, TN, AK, AN, and AP at Chajintai Ranch were all significantly higher than those at Bahuta Sumu (*p* < 0.05), indicating distinct nutrient supply conditions that provided favorable conditions for comparing rhizosphere AMF community responses of *C. pauciflorus* and co-occurring plants under different phosphorus conditions. It should be noted that the two sites differed not only in phosphorus supply but also showed concurrent changes in nitrogen, potassium, and organic matter, indicating that AMF community differences between the two field sites resulted from the combined effects of multiple soil factors.

At each site, six 10 m × 10 m plots were randomly established, with spacing of more than 10 m between plots. Within each plot, five subplots dominated by *C. pauciflorus* were selected. In each subplot, three plants were sampled in an S-shaped pattern. Root systems and attached soil were excavated to a depth of approximately 20 cm with a shovel. Soil adhering to the root surface was collected as rhizosphere soil. Rhizosphere soil and root samples from the five subplots within each plot were pooled separately into one composite sample. Thus, two composite samples (one rhizosphere soil and one root sample) were obtained per plot, yielding 12 samples per site. *Setaria viridis* was sampled following the same protocol from the same subplots where it co-occurred with *C. pauciflorus* (Table 2). All rhizosphere soil samples were passed through a 2 mm sieve, mixed, and placed in sterile bags. All root samples were also mixed and placed in sterile bags, kept in a cool box, and transported to the laboratory. AMF community diversity in root and rhizosphere soil samples was analyzed using high-throughput sequencing to compare differences in AMF communities between *C. pauciflorus* and *S. viridis* and to identify AMF taxa specifically enriched by *C. pauciflorus*.

### 4.2. Determination of AMF Diversity

AMF diversity was analyzed by AMF-specific 18S rRNA gene amplicon sequencing. The analysis was performed by Shanghai Majorbio Bio-pharm Technology Co., Ltd. (Shanghai, China). DNA was extracted from root and rhizosphere soil samples using the FastPure Soil DNA Isolation Kit (YH-Soil, Majorbio Yuhua, Shanghai, China). DNA quality was verified by 1% agarose gel electrophoresis. The AMF 18S rRNA gene region was amplified using primers AMV4-5NF/AMDGR (sequences: 5′-AAGCTCGTAGTTGAATTTCG-3′ and 5′-CCCAACTATCCCTATTAATCAT-3′). PCR was performed in a 20 μL reaction mixture containing 10 μL 2× Pro Taq Master Mix, 0.8 μL of each primer (5 μM), 1 μL template DNA (10 ng/μL), and ddH_2_O to a final volume of 20 μL. The thermal cycling conditions were as follows: 95 °C for 3 min; 25 cycles of 95 °C for 30 s, 55 °C for 30 s, and 72 °C for 45 s; followed by 72 °C for 10 min. Sterile water was used as a negative control. PCR products were pooled in equal amounts, purified using the AxyPrep DNA Gel Extraction Kit, and sequenced on the Illumina NovaSeq 6000 PE300 platform (Illumina, San Diego, CA, USA). Sequencing data were processed on the Majorbio Cloud Platform (Majorbio Yuhua, Shanghai, China) for merging, quality control, OTU clustering, and chimera removal.

### 4.3. Determination of Soil Physicochemical Properties

At each site, soil was collected from six plots. Within each plot, three soil cores (0–10 cm) were collected with a soil auger and mixed into one composite sample, yielding six composite samples per site (*n* = 6). The mean of the six composite samples was used to represent the soil physicochemical properties at each site. Soil pH was measured with a pH meter at a water:soil ratio of 5:1. Total nitrogen (TN) was determined by the Kjeldahl method. Total phosphorus (TP) was measured by perchloric acid–sulfuric acid digestion followed by molybdenum-antimony colorimetry. Available phosphorus (AP) was extracted with 0.5 mol/L NaHCO_3_ and determined by molybdenum-antimony colorimetry. Available nitrogen (AN) was determined by alkaline hydrolysis diffusion. Available potassium (AK) was extracted with ammonium acetate and measured by flame photometry [33,34].

### 4.4. Plant Inoculation Experiment

Surface aeolian sandy soil was collected from bare areas at the Bahuta Sumu site. The soil was passed through a 2 mm sieve to remove gravel and plant debris, washed repeatedly to remove organic fragments, autoclaved at 121 °C for 21 min, and air-dried for use as the potting substrate.

Based on microbiome analysis, *Funneliformis mosseae* (Fm), a representative species of the genus *Glomus* that was significantly enriched by both plants, and *Diversispora epigaea* (De), a representative of the genus *Diversispora* enriched in *C. pauciflorus* roots, were selected as inoculants. Both inoculants were root–soil mixtures with spore densities of 156 spores per 10 g (Fm) and 143 spores per 10 g (De), respectively. They were purchased from the Root Biology Institute of Yangtze University.

The pot experiment consisted of five phosphorus treatments. Phosphorus addition rates were based on dry substrate weight (g P per 1.2 kg dry substrate). The high-phosphorus control treatment (HP) received 0.05 g P per 1.2 kg as KH_2_PO_4_. At the low-phosphorus level of 0.005 g P per 1.2 kg substrate, four phosphorus forms were applied: KH_2_PO_4_ (LP), aluminum phosphate (AlPO_4_, LAl), phytic acid (C_6_H_18_O_24_P_6_, LZ), and hydroxyapatite (Ca_5_(PO_4_)_3_(OH), LQ). The concentrations used in the low-phosphorus treatments were close to the lower limit of the background soil available phosphorus level (5–46 mg/kg) in *C. pauciflorus* invasion habitats in the Horqin Sandy Land, aiming to simulate the low-phosphorus and heterogeneous phosphorus speciation conditions of the region [35,36].

Sterilized fine sand and vermiculite were mixed at a 1:1 (*v*/*v*) ratio as the growth substrate. Each pot contained 1.2 ± 0.1 kg of mixed substrate. Five grams of Fm or De inoculum and the corresponding phosphorus treatment were thoroughly mixed with the substrate, and the mix was placed into plastic pots (top diameter 12.8 cm, height 16.6 cm). Non-inoculated controls were included for each phosphorus treatment. Eight replicates were prepared for each phosphorus × AMF treatment combination.

Uniform, plump *C. pauciflorus* burs were surface-sterilized in 0.5% NaClO for 15 min, rinsed three times with sterile water, and sown in pots (6 burs per pot). Two weeks after emergence, seedlings were thinned to three uniform plants per pot, and pots were maintained as uniform as possible both within and across treatments. Plants were grown in a greenhouse under natural light supplemented with artificial lighting (14 h/10 h light/dark photoperiod, light intensity approximately 600 μmol·m^−2^·s^−1^) at an average temperature of 27 °C. To ensure normal growth, phosphorus-free Hoagland nutrient solution (200 mL per pot) was applied every 7 d starting two weeks after thinning, for a total of seven applications. The nutrient solution composition was: 607.2 mg/L KNO_3_, 40 mg/L NH_4_NO_3_, 241 mg/L MgSO_4_, 36.7 mg/L FeNaEDTA, 0.83 mg/L KI, 6.2 mg/L H_3_BO_3_, 22.3 mg/L MnSO_4_, and 8.6 mg/L ZnSO_4_. Sterile water was added according to substrate moisture content to maintain 80% of maximum field capacity. Pots were randomly rotated weekly to minimize microenvironmental differences within the greenhouse.

### 4.5. Measurement of Growth and Colonization Indices

One week after the seventh application of phosphorus-free nutrient solution, *C. pauciflorus* fruits had begun to turn yellow and naturally abscise, indicating the end of fruit development. Five pots were randomly selected from each treatment for growth and biomass measurements, while the remaining three pots were used for mycorrhizal colonization analysis. For each of the five selected pots, plant height, tiller number, spike number (total fruiting spikes per plant), and fruit number (number of burs on the main rachis) were measured. All green leaves from each plant were placed flat on a white background board, photographed with a digital camera, and leaf area was calculated using Adobe Photoshop 2025 software. Plants were then carefully removed from the pots and separated into roots, stems, leaves, and fruits. Samples were oven-dried at 105 °C for 30 min, then at 80 °C to constant weight. Dry biomass of each organ was weighed using an analytical balance to the nearest 0.001 g. All morphological indices and biomass values were represented as the average of three plants per pot, with five replicate pots per treatment (*n* = 5). For mycorrhizal analysis, roots from the three remaining pots were cleared in KOH, stained with acid fuchsin (concentration, time), and examined under a microscope to record vesicle and hyphal colonization [37]. Colonization rates were calculated as the percentage of colonized root segments or grid intersections for each of the three replicate samples per treatment (*n* = 3).

### 4.6. Data Processing and Analysis

Data were compiled using Microsoft Excel 2019. Statistical analyses were performed using SPSS 19.0. Growth data and α-diversity indices were analyzed using one-way ANOVA followed by LSD multiple-comparison tests (*p* < 0.05). Soil physicochemical properties were compared using independent-samples t-tests. AMF colonization rates were analyzed using the Kruskal–Wallis H test, with post-hoc pairwise comparisons performed using the Mann–Whitney U test. α-Diversity indices (ACE, Chao1, Shannon, and Simpson) were calculated, and β-diversity was assessed using NMDS ordination based on Bray–Curtis distances, with differences among groups tested by ANOSIM (999 permutations). All data were presented as mean ± standard error (SE). Differences were considered statistically significant at *p* < 0.05.

## 5. Conclusions

This study investigated the differences in AMF community composition under different soil phosphorus conditions in the Horqin Sandy Land through combining field surveys and pot inoculation experiments, and evaluated the growth-promoting effects of *Funneliformis mosseae* and *Diversispora epigaea* on the invasive plant *Cenchrus pauciflorus* under different phosphorus supply forms.

At the community level, soil phosphorus condition was closely related to AMF community composition and diversity. AMF α-diversity was higher at the high-phosphorus site (Chajintai Ranch) than at the low-phosphorus site (Bahuta Sumu). AMF diversity in rhizosphere soil was generally higher than that in roots. Glomeraceae and *Glomus* were the dominant taxa in all samples, while *Diversispora* and *Acaulospora* were relatively enriched in the roots of *C. pauciflorus* at the low-phosphorus site, suggesting that *C. pauciflorus* may selectively enrich specific AMF taxa under nutrient-limited conditions. Nevertheless, because phosphorus, nitrogen, potassium, and other indices changed concurrently between the two field sites, the independent effect of phosphorus could not be fully disentangled.

At the functional validation level, the colonization rates and growth-promoting effects of the two AMF species showed clear dependence on phosphorus treatment and species specificity. Under the high-phosphorus treatment, AMF inoculation did not promote the growth of *C. pauciflorus*, and several growth indices were lower than those in the non-inoculated control. Under the low-phosphorus treatments with insoluble phosphorus sources, both AMF species promoted growth and biomass accumulation in *C. pauciflorus*. *D. epigaea* exhibited stronger growth-promoting effects than *F. mosseae* under the LAl and LQ treatments, and its vesicle colonization rate was significantly higher than that of *F. mosseae* in multiple treatments, indicating that *D. epigaea* had stronger root colonization ability under several phosphorus treatments. The two AMF species therefore differed in their colonization and growth-promoting effects.

Taken together, the *Diversispora* genus enriched in the field survey and its representative species *D. epigaea* validated in the pot experiment showed consistent functional effects, suggesting that the enrichment of this genus by *C. pauciflorus* under low-phosphorus conditions may be confer functional advantages. The successful invasion of *C. pauciflorus* in the Horqin Sandy Land may partly attributed to its ability to enrich specific AMF taxa in low-phosphorus habitats, thereby enhancing the acquisition of sparingly soluble phosphorus through belowground symbiotic interactions. This enhanced phosphorus acquisition may be subsequently translated into advantages in growth and reproduction. This study provides evidence for the “enhanced mutualism” hypothesis by examining a sandy-land invasive plant and offers theoretical support for understanding the phosphorus adaptation strategies of *C. pauciflorus* from a belowground symbiotic perspective.

## Figures and Tables

**Figure 1:**
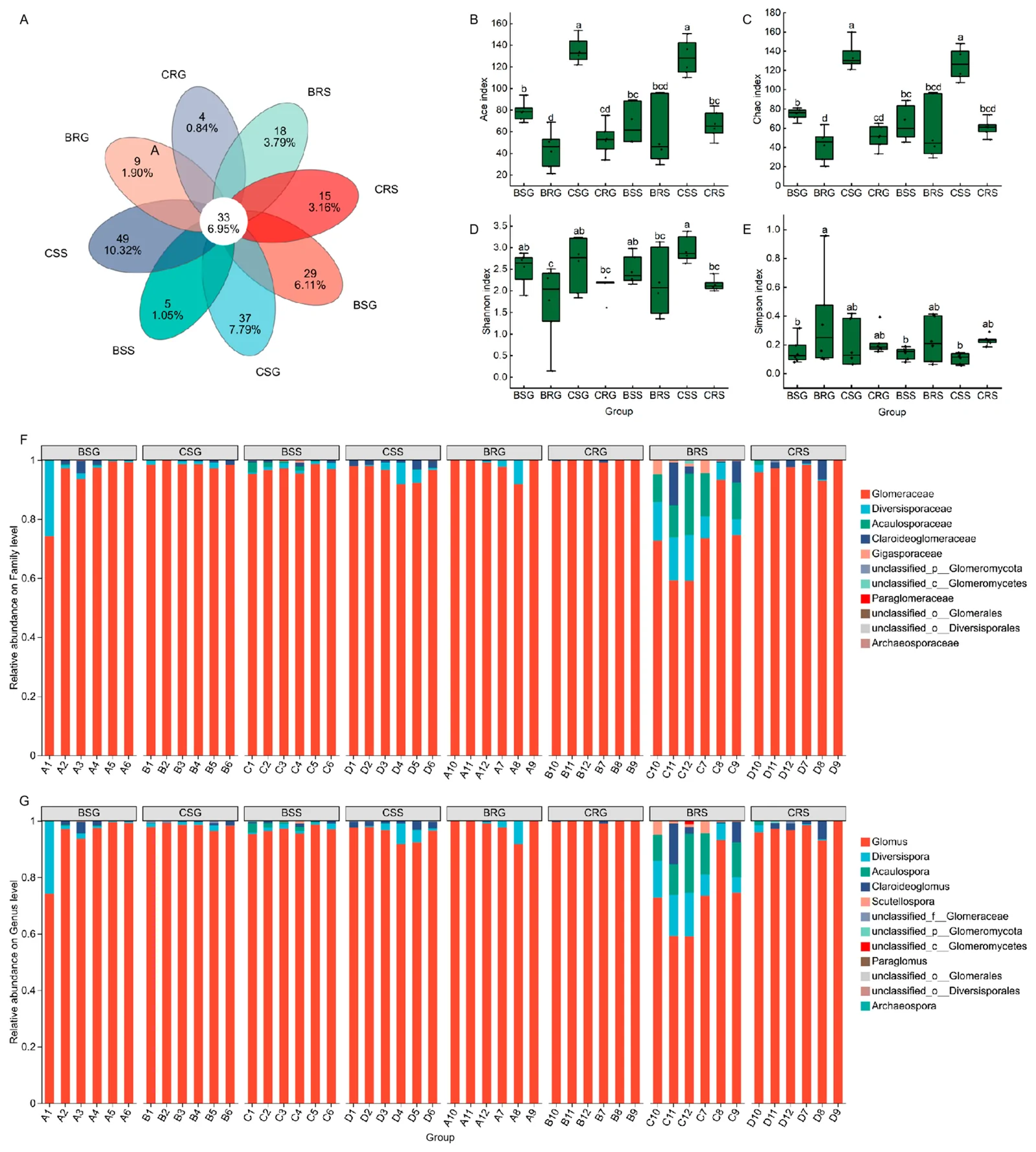
AMF OTU distribution, α-diversity, and taxonomic composition at the family and genus levels across sample groups. (**A**) Venn diagram of AMF OTUs among sample groups; (**B**–**E**) Ace index (**B**), Chao index (**C**), Shannon index (**D**), and Simpson index (**E**) across sample groups. The horizontal lines in the boxplots represent medians, and lowercase letters indicate significant differences among groups (*p* < 0.05); (**F**) relative abundance of AMF at the family level; (**G**) relative abundance of AMF at the genus level. Unclassified sequences were defined as those that failed to match any sequence in the database at a 70% confidence leve. Sample codes: BSG, rhizosphere soil of *S. viridis* from Bahuta Sumu; BRG, roots of *S. viridis* from Bahuta Sumu; CSG, rhizosphere soil of *S. viridis* from Chajintai Ranch; CRG, roots of *S. viridis* from Chajintai Ranch; BSS, rhizosphere soil of *C. pauciflorus* from Bahuta Sumu; BRS, roots of *C. pauciflorus* from Bahuta Sumu; CSS, rhizosphere soil of *C. pauciflorus* from Chajintai Ranch; CRS, roots of *C. pauciflorus* from Chajintai Ranch; *n* = 6 for each group.

**Figure 2:**
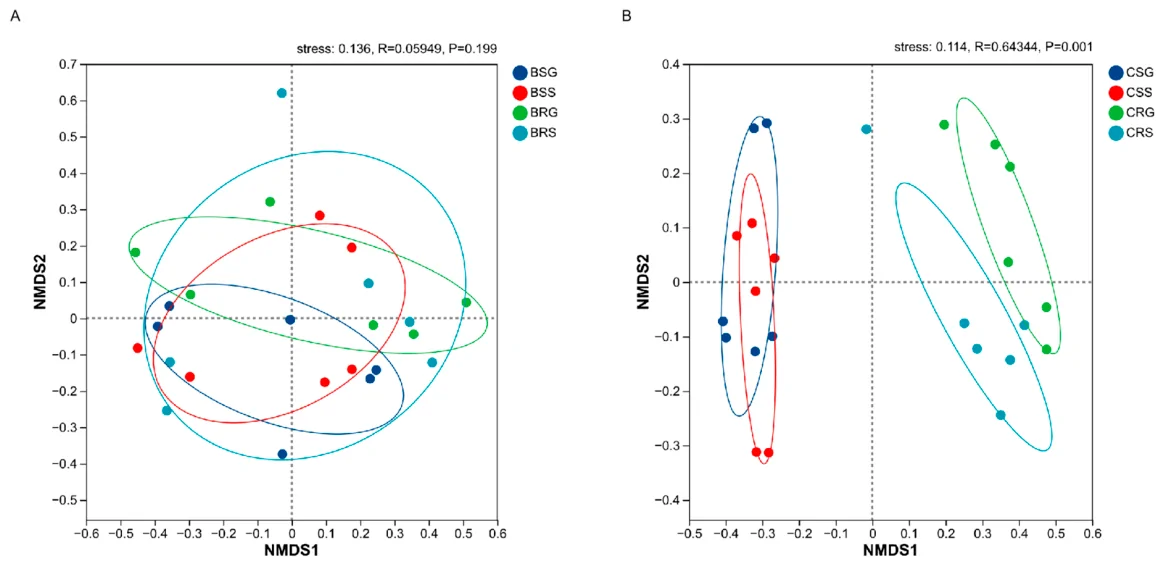
AMF community structure. (**A**) NMDS ordination of AMF communities in the four sample groups from Bahuta Sumu; (**B**) NMDS ordination of AMF communities in the four sample groups from Chajintai Ranch.

**Figure 3:**
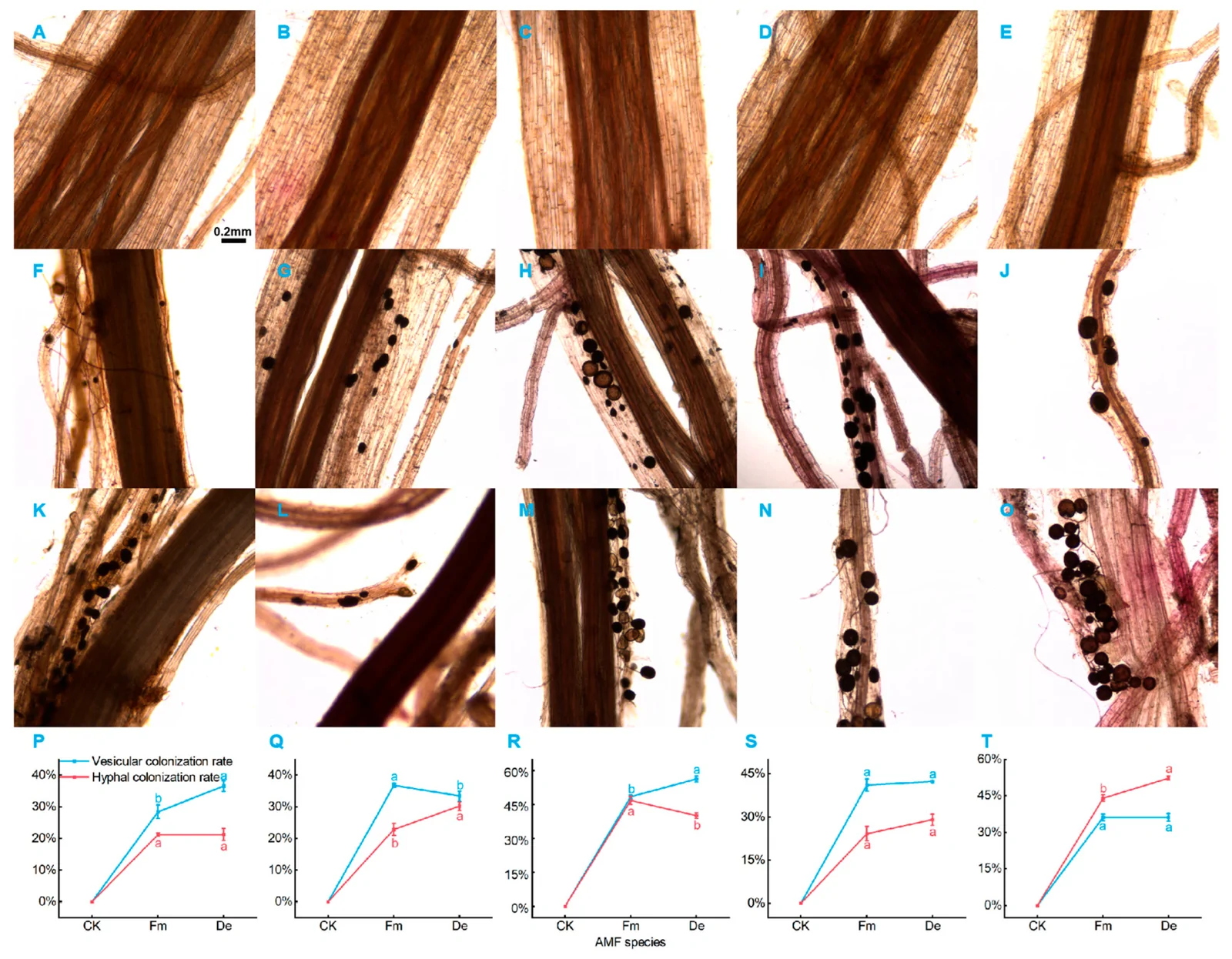
Micrographs of AMF colonization and vesicle and hyphal colonization rates in *Cenchrus pauciflorus* roots under AMF inoculation and phosphorus treatments. (**A**–**E**) Micrographs of non-inoculated controls under different phosphorus treatments; (**F**–**J**) micrographs of roots inoculated with *Funneliformis mosseae* (Fm); (**K**–**O**)micrographs of roots inoculated with *Diversispora epigaea* (De); (**P**–**T**) vesicle and hyphal colonization rates under different phosphorus treatments. Each column denotes one phosphorus treatment; treatments are arranged from left to right as HP, LP, LAl, LQ, and LZ. Lowercase letters indicate significant differences in colonization rates between Fm and De under the same phosphorus treatment (*p* < 0.05).

**Figure 4:**
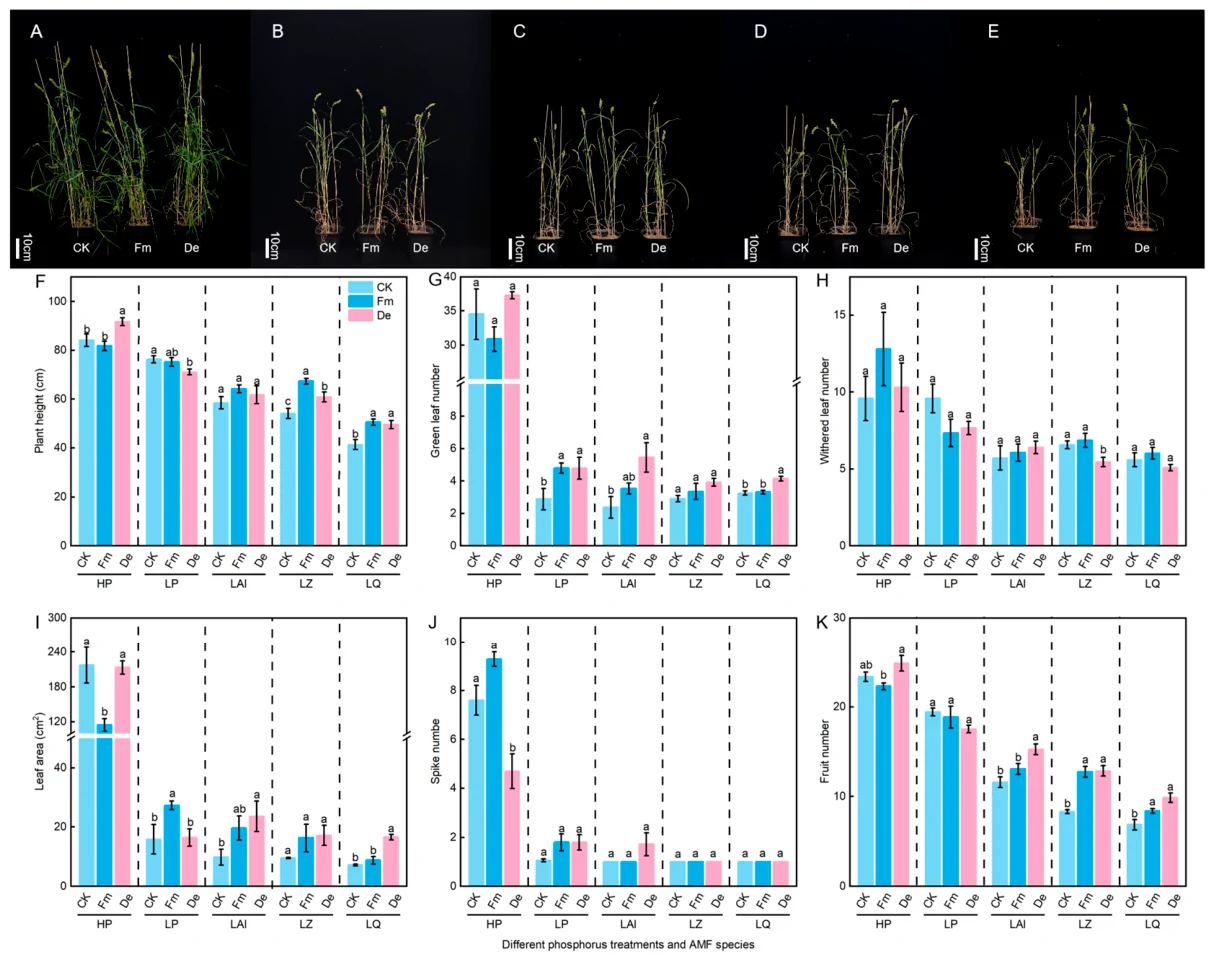
Growth indices of *Cenchrus pauciflorus* under AMF inoculation and phosphorus treatments. (**A**–**E**) Growth photographs of *C. pauciflorus* inoculated with different AMF species under different phosphorus treatments (A, HP; B, LP; C, LAl; D, LZ; E, LQ); (**F**) plant height; (**G**) green leaf number; (**H**) withered leaf number; (**I**) leaf area; (**J**) spike number; (**K**) fruit number. Lowercase letters indicate significant differences among AMF treatments under the same phosphorus treatment (*p* < 0.05). *n* = 5 for each treatment; error bars represent standard error (SE).

**Figure 5:**
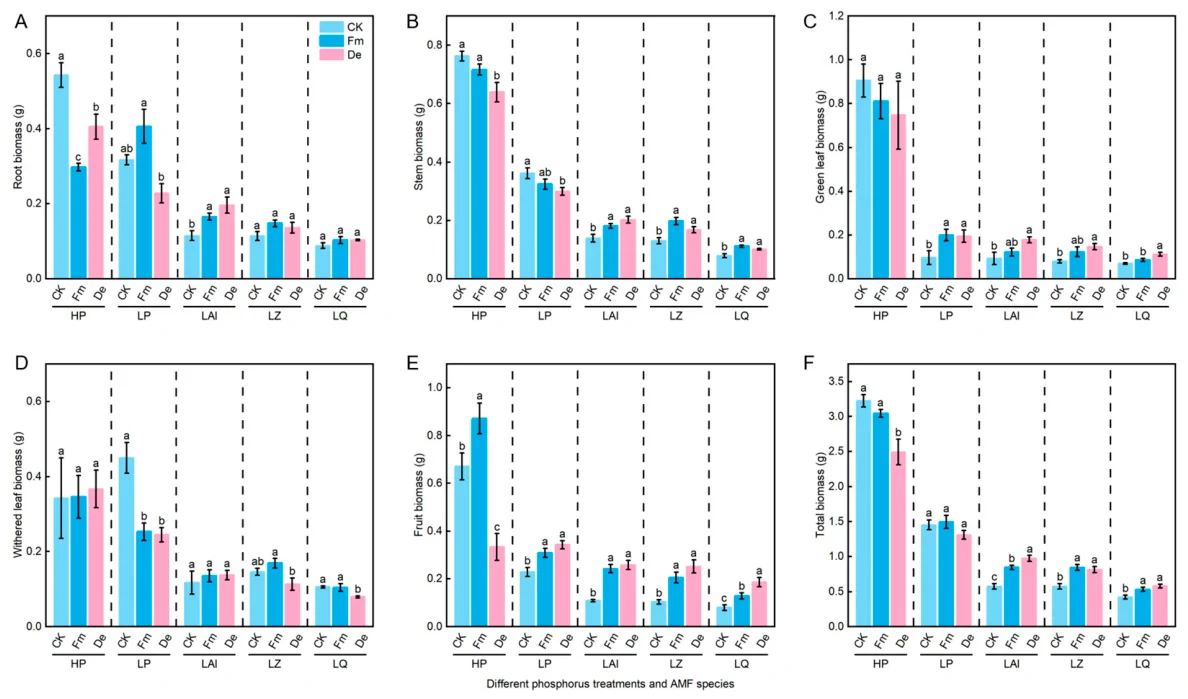
Biomass of *Cenchrus pauciflorus* under different AMF inoculation and phosphorus treatments. (**A**–**F**) Root biomass (**A**), stem biomass (**B**), green leaf biomass (**C**), withered leaf biomass (**D**), fruit biomass (**E**), and total biomass (**F**) under the different phosphorus treatments. Lowercase letters indicate significant differences in biomass among AMF treatments under the same phosphorus treatment (*p* < 0.05). *n* = 5 for each treatment; error bars represent standard error (SE).

**Table 1:** Soil physicochemical properties at the two sampling sites.

Soil Physicochemical Properties	Bahuta Sum	Chajintai Ranch	*p* Value
pH	8.62 ± 0.04	7.98 ± 0.08	<0.001 ***
TP (mg/kg)	633.94 ± 47.31	54.23 ± 6.50	<0.001 ***
TN (mg/kg)	1217.22 ± 11.43	152.56 ± 20.30	<0.001 ***
AK (mg/kg)	103.79 ± 7.71	21.68 ± 0.98	<0.001 ***
AP (mg/kg)	9.72 ± 2.02	0.50 ± 0.10	0.006 **
AN (mg/kg)	52.05 ± 4.76	12.14 ± 1.50	<0.001 ***

Note: TP, total phosphorus; TN, total nitrogen; AK, available potassium; AP, available phosphorus; AN, available nitrogen. Values are means ± standard error (*n* = 6). * *p* < 0.05, ** *p* < 0.01, *** *p* < 0.001.

**Table 2:** Sample information and grouping of rhizosphere soil and root samples from two sites.

Site	Bahuta Sum				Chajintai Ranch			
Sample part	Rhizosphere soil		Root		Rhizosphere soil		Root	
Species	*Setaria viridis*	*Cenchrus pauciflorus*	*Setaria viridis*	*Cenchrus pauciflorus*	*Setaria viridis*	*Cenchrus pauciflorus*	*Setaria viridis*	*Cenchrus pauciflorus*
Group label	BSG	BSS	BRG	BRS	CSG	CSS	CRG	CRS
Sample number	A-1	C-1	A-7	C-7	B-1	D-1	B-7	D-7
	A-2	C-2	A-8	C-8	B-2	D-2	B-8	D-8
	A-3	C-3	A-9	C-9	B-3	D-3	B-9	D-9
	A-4	C-4	A-10	C-10	B-4	D-4	B-10	D-10
	A-5	C-5	A-11	C-11	B-5	D-5	B-11	D-11
	A-6	C-6	A-12	C-12	B-6	D-6	B-12	D-12

## Data Availability

The raw data supporting the conclusions of this article will be made available by the authors on request.
